# Case report: Characterization of a rare pathogenic variant associated with loss of COL3A1 expression in vascular Ehlers Danlos syndrome

**DOI:** 10.3389/fcvm.2022.939013

**Published:** 2022-10-11

**Authors:** Janvie Manhas, Lov Raj Lohani, Ashikh Seethy, Uma Kumar, Shivanand Gamanagatti, Sudip Sen

**Affiliations:** ^1^Department of Biochemistry, All India Institute of Medical Sciences, New Delhi, India; ^2^Department of Rheumatology, All India Institute of Medical Sciences, New Delhi, India; ^3^Department of Radiodiagnosis, All India Institute of Medical Sciences, New Delhi, India

**Keywords:** vascular Ehlers Danlos syndrome, COL3A1 pathogenic variant, clinical fibroblast testing, exome sequencing, stroke, hepatic artery dissection

## Abstract

The vascular subtype of Ehlers Danlos Syndrome (vEDS) is a rare connective tissue disorder characterized by spontaneous arterial, bowel or organ rupture. The diagnosis of vEDS is established in a proband by identification of a heterozygous pathogenic variant in the alpha-1 gene of type III collagen (*COL3A1*) by molecular analysis. In this report, we present a case of vEDS with life threatening, spontaneous arterial dissections in association with an uncharacterized rare variant of *COL3A1, exon19:c.1340G* > *A*. Primary culture of patient skin fibroblasts followed by immunofluorescence revealed a complete absence of COL3A1 protein expression as well as altered morphology. Electron microscopy of the cultured fibroblasts showed abnormal vacuoles in the cytoplasm suggestive of a secretory defect. In this study, we have performed functional characterization of the *COL3A1 exon19:c.1340G* > *A* variant for the first time and this may now be classified as likely pathogenic in vEDS.

## Introduction

Vascular Ehlers Danlos Syndrome (vEDS; OMIM130050) is a rare connective tissue disorder caused by mutations in the alpha-1 chain of type III collagen (COL3A1) polypeptide leading to serious risk of arterial or organ rupture and premature death (1). It is dominantly inherited in 50% cases and the other 50% present with *de novo* pathogenic, somatic variants in *COL3A1* (2). The most common *COL3A1* pathogenic variant is a heterozygous missense substitution for glycine in the (Gly-X-Y) repeating sequence of collagen triple helix which disrupts the assembly of type III homotrimeric collagen (3). This leads to defective type III collagen synthesis and assembly, manifesting as loss of mechanical strength in arteries and other hollow organs. The natural course of vEDS and associated clinical phenotype of patients are both reported to be influenced by the type of *COL3A1* variant (3, 4). Therefore, establishing a diagnosis of vEDS should ideally always include molecular genetic testing for *COL3A1* pathogenic variants and biochemical analysis to ascertain the pathogenicity of unreported variants (5).

Due to limited genetic testing and unavailability of clinical fibroblast culture testing, the prevalence and burden of vEDS in India is underestimated and underreported. vEDS is defined based on one major and several minor diagnostic criteria (1) which highlight the plethora of different physical signs that may constitute the clinical phenotype and cause confusion during diagnosis. Considering the unpredictable, life threatening complications of vEDS with a low median survival of around 48 years (2), it becomes imperative to employ genetic testing early during the management of a suspected vEDS patient especially when the phenotype is indistinguishable from other inherited connective tissue disorders.

## Case description

**FIGURE 1:**
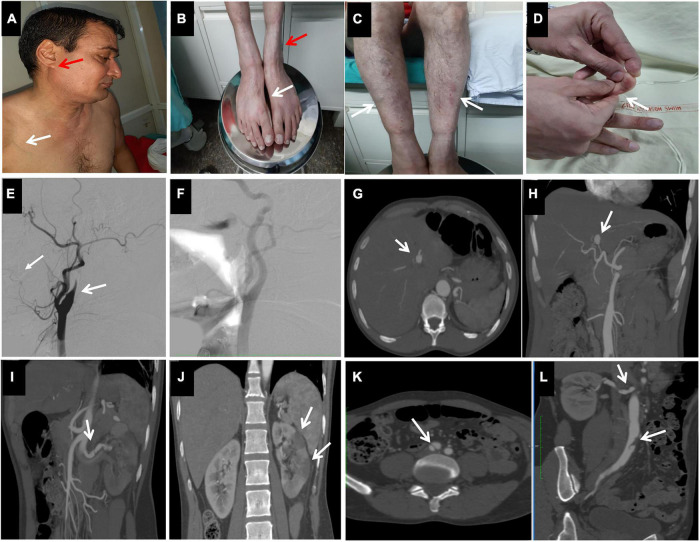
Observed clinical features of the patient, **(A):** Thin Nose, lobe less ear (red arrow), thin skin with visible chest veins (white arrow), **(B):** Acrogeria (red arrow) with club foot (white arrow), **(C):** Easy bruising and thin skin, varicose veins, **(D):** Hypermobility of small joints (shown in distal phalange of index finger). Digital subtraction angiography images of bilateral common carotid arteries showing dissection of left internal carotid artery **(E,F)**. Right internal carotid artery was normal. Axial **(G)** and coronal **(H)** maximum intensity projection (MIP) CT images showing aneurysmal dilatation of left hepatic artery. Coronal MIP images **(I,J)** of contrast enhanced CT show dissection involving left renal artery with multiple infarcts of renal parenchyma. Axial **(K)** and coronal **(L)** MIP CT images show dissection involving right renal artery and right common iliac artery.

A 48-year-old male with no past history of any relevant disease presented with the sudden onset of severe headache followed by right sided hemiparesis. On examination the patient had a thin face with prominent nose and lobeless ears (Figure 1A) along with fragile and thin skin over the extremities suggestive of Acrogeria (Figure 1B). Presence of varicose veins with some ecchymotic patches were observed over the lower limbs (Figure 1C). Chest veins were also found to be prominent (Figure 1A). Hypermobility of small joints of the hands (Figure 1D) and clubbing of feet were observed (Figure 1B). However, hyper elasticity of skin and marfanoid features were not present. Bilateral pitting edema was present in the lower limbs. All other vital parameters were within normal range.

Digital Subtraction Angiography (DSA) was suggestive of dissection with sub occlusive narrowing in the left internal carotid artery in the skull base region (Figures 1E,F). Patient was started on anticoagulation therapy with vitamin K antagonist (VKA) and 5 mg of warfarin, dose titrated to maintain a target INR (International Normalized Ratio) of 2–3. Hemiparesis gradually subsided and warfarin was stopped.

Patient was asymptomatic for 1 year followed by the sudden onset of upper abdominal pain. Computed tomography (CT) angiography showed aneurysmal dilatation of left hepatic artery (Figures 1G,H) along with multiple arterial dissections involving left renal artery (Figures 1I,J) and multiple infarcts in right renal artery and proximal right common iliac artery (Figures 1K,L). The patient also developed elevated blood pressure which was a new symptom and was managed using anti-hypertensives. A rise in the level of the inflammatory marker, C-reactive protein was observed in serum. Nerve conduction study and nerve biopsy were suggestive of axonal and demyelinating neuropathy in both lower legs. Blood tests for vasculitis markers including antinuclear antibody, perinuclear and cytoplasmic antineutrophil cytoplasmic antibodies (myeloperoxidase and proteinase) were found to be negative. The patient was negative for HIV, hepatitis B and C. Within a few days our patient presented with another episode of acute abdominal pain with bilateral subcostal tenderness. He was diagnosed with hemoperitoneum due to Hepatic Artery rupture with ischemic liver changes, for which aneurysmal coiling was performed. There was no history of weight loss, fever, hemoptysis or melena. Laboratory test results are shown in Supplementary Table 1.

### Whole exome sequencing analysis reveals a likely pathogenic *COL3A1* variant of uncertain significance

DNA was isolated from the peripheral blood sample of the patient and sent for whole exome sequencing (WES) to identify the cause of the disease. A total of 34058 variants were called after filtering for the depth of sequencing (minimum 10), of which 9257 were exonic variants, including single nucleotide variants and short indels. Exclusion of synonymous variants and filtering for minor allele frequency of <5% retained 854 variants. Once the non-pathogenic variants were removed using SIFT, Polyphen2-HDIV and Mutation Assessor predictions, 548 variants remained (Supplementary Data 1). After inclusion of 28 variants which affected splicing, there were 576 pathogenic variants across 392 different genes, of which 154 were found to be associated with a phenotype in OMIM.

On further evaluation based on the patient’s phenotype of ‘blood vessel abnormalities AND hypermobility’ in OMIM, *COL3A1 exon19:c.1340G* > *A* [chr2:g.188994587G > A,NM_000090.3:c.1340G>A, NM_000090.3(COL3A1_i001):p.(Gly447Asp), all descriptions are based on hg38] variant leading to the substitution of Aspartic acid for Glycine at codon 447 (p.G447D) in type III collagen α1 polypeptide was identified. The variant attributes according to the American College of Medical Genetics and Genomics (ACMG) and the Association for Molecular Pathology (AMP) guidelines (6) for the interpretation of sequence variants were PM2, PP2, and PP3. Visualization of this variant in Integrative Genome Viewer (IGV) suggested heterozygosity (Supplementary Data 1 in Supplementary Figure 1). This variant has been previously found to be associated with vascular subtype of Ehlers Danlos syndrome in one case report (7). Since this variant has not been reported in the 1000 genomes (8), Exome Aggregation Consortium (9) (ExAC) and other databases (10) and no functional data was available regarding this variant, we further proceeded to analyze the pathogenic basis of this variant using molecular techniques.

### The Exon19:c. *1340G* > *A* variant leads to absence of COL3A1 protein in skin fibroblasts

**FIGURE 2:**
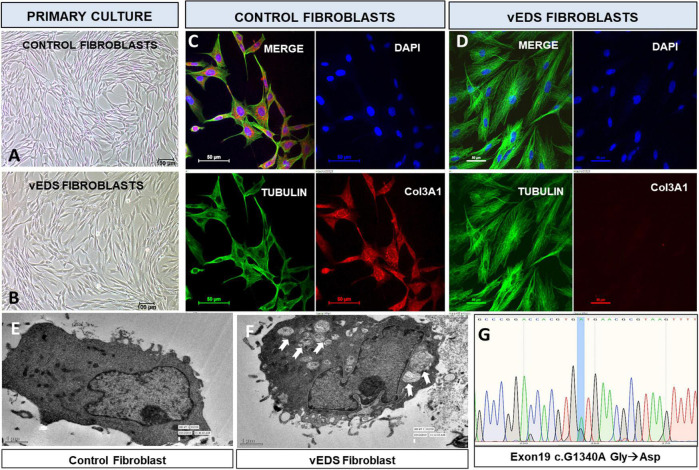
Primary culture of skin derived fibroblasts from **(A)** age matched control **(B)** suspected vEDS patient. Immunofluorescence analysis of fibroblasts show **(C)** presence of Col3A1(Red) control fibroblasts whereas **(D)** patient fibroblasts show absence of Col3A1. Transmission electron microscopy of **(E)** control fibroblasts with normal morphology, **(F)** vEDS fibroblasts with altered morphology, arrows show intracytoplasmic lamellar vacuolar bodies. **(G)** Sequences of wild-type (1340-G) and mutant (1340-A) COL3A1 cDNA derived from patient skin fibroblasts. As vEDS patient is heterozygous for COL3A1 c.1340G > A mutation, sequencing of the region harboring the mutation results in detection of both guanine and adenine at position 1340. The G > A mutation leads to an exchange from glycine to aspartic acid at the protein level.

Patient and control fibroblasts were cultured from skin biopsy (11) (Figures 2A,B) and analysis of protein expression of COL3A1 was done using immunofluorescence. There was a complete absence of COL3A1 production in the patient fibroblasts compared to the control fibroblasts (Figures 2C,D). The morphology of patient fibroblasts was also observed to be altered as compared to the control fibroblasts. Fibroblasts derived from skin biopsy of patient with exon19:c. *1340G* > *A* variant were larger in size as compared to the control fibroblasts (Figures 2C,D). It has been previously reported that dermal fibroblasts from vEDS have defective collagen biosynthesis/processing, endoplasmic reticulum homeostasis/protein folding, disorganized ECM interactions and may acquire a myofibroblast like phenotype (12). In order to investigate the altered morphology, we performed a Transmission electron microscopy (TEM) analysis. TEM has previously been suggested to be a useful addition to the repertoire of diagnostic tests in vEDS (13). Previous studies have reported an inefficient secretion of mutant collagen molecules which can lead to altered structural anatomy of the endoplasmic reticulum and unfolded protein response (14). We observed lamellar vacuolar bodies in the cytoplasm of vEDS fibroblasts which were not present in control fibroblasts (Figures 2E,F). On in-depth observational analysis, these bodies appeared to be “Amphisomes,” organelles of the autophagy pathway resulting from fusion of endosomes and autophagosomes (15). This may explain the altered morphology and size of vEDS fibroblasts.

### Sanger sequencing

Genomic DNA was also extracted from cultured dermal fibroblasts from patient and age-matched control and the *COL3A1 exon19:c.1340G* > *A* variant obtained from Whole exome sequencing was confirmed by Sanger sequencing (Figure 2G). All methodology provided in Supplementary Data 2.

## Discussion

Diagnosis of vEDS based on clinical diagnostic criteria alone is often difficult due to significant overlap in clinical presentation with other connective tissue disorders and arteriopathies. vEDS diagnosis should be suspected in individuals presenting with spontaneous ruptures of arteries, uterus or bowel at a young age. Careful interpretation of genetic testing results and variant assessment according to the ACMG/AMP guidelines (6) is essential to confirm the diagnosis, as all COL3A1 variants are not pathogenic (16). Additionally, clinical fibroblast testing and functional analysis can help to accurately report pathogenicity of novel sequence variants.

### Classification of *COL3A1* exon19:c.1340G > A as likely pathogenic variant

About 705 unique likely pathogenic/pathogenic *COL3A1* variants have been reported, which are listed in the *Leiden Open Variation Database* (see https://databases.lovd.nl/shared/genes/COL3A1). Missense mutation involving substitution of a glycine amino acid in the triple helix is the most common type of mutation reported in the database. The second most common type of mutation is a *COL3A1* RNA splicing mutation. Large deletions or insertions in *COL3A1* are relatively uncommon in vEDS. Most patients with vEDS develop major complications before the age of 30 years. Autosomal dominant inheritance of vEDS shows a penetrance approaching 100% but may also vary according to the age of presentation. Haploinsufficient patients have a lower penetrance of ∼50% and were observed to survive 10–15 years longer than individuals harboring RNA splicing or glycine mutations (17). Interestingly, a missense mutation with substitution of a valine or an aspartic acid for a glycine was reported to have a poorer prognosis than a substitution of a serine (17). Although the phenotype of patients with amino acid substitutions in COL3A1 may partially overlap with of some haploinsufficient patients (18), there is considerable evidence for genotype-phenotype correlation (3, 16, 17) in vEDS which may be critical for screening and lifelong management of the affected individual and family members.

Our patient presented with stroke along with cranial and abdominal visceral arteries’ dissections and rupture. Patient was hypertensive, had an elevated C-reactive protein (CRP) and neuropathy which lead to an initial suspicion of Polyarteritis Nodosa (PAN) (19). However, he did not show constitutional symptoms of fever, fatigue, weight loss, muscle/joint pains, radiological findings or other features defined by American College of Rheumatology classification criteria for PAN. The clinical presentation also favored the differential diagnosis of vEDS, but the presenting age of 48 years was not consistent with this diagnosis. The presence of multiple clinical findings namely acrogeria, progeria, lobeless ear, hypermobile small joints of hands, thin fragile skin, foot clubbing and multiple arterial dissections favored the diagnosis of vEDS. Patient had renal infarcts following renal artery dissection which could have contributed to the rise in blood pressure. It has been reported that delayed clearance of thrombi as well as the vascular insult to arteries in vEDS may lead to a rise in inflammatory markers (20), as observed in this case. Presence of axonal polyneuropathy has also been reported before in vEDS (21, 22). As there was no family history of EDS or related syndromes, in order to establish the diagnosis in a proband it was imperative to perform molecular genetic testing to identify and characterize this variant (2). Corticosteroids are used in PAN to reduce inflammation but they have been reported to trigger arterial dissections by elevating blood pressure and increasing blood vessel fragility by its inhibitory effect on collagen formation and connective tissue strength (23). Therefore, the patient was managed symptomatically till the genetic testing results were obtained.

Whole exome sequencing revealed a rare, uncharacterized variant of *COL3A1* gene, exon19:c.1340G > A variant. A molecular diagnostic study was carried out to confirm if this variant was responsible for the vEDS phenotype. Dermal Fibroblast culture was established using skin biopsy from the patient and age matched control and COL3A1 protein expression was studied using immunofluorescence. The COL3A1 expression was observed to be absent in the exon19:c.1340G > A variant (Figures 2C,D). This suggests the expression of a mutant collagen with a dominant negative effect leading to a possible degradation after binding with the wild type collagen molecule. The presence of the variant was further confirmed by Sanger sequencing of the PCR amplicon obtained by using DNA extracted from cultured fibroblasts as the template and primers flanking the site of the variant (Figure 2G). We also observed 2 more non-synonymous variants of *COL3A1* (exon 30:c.2092G > A and exon 50:c.4059T > G) during the data analysis of the WES. However, based on their high minor allele frequencies, these were found to be non-deleterious (Table 1). Transmission Electron Microscopy (TEM) studies of the dermal fibroblasts suggested that exon19:c.1340G > A variant is associated with altered endoplasmic reticular morphology in vEDS which may contribute to the disease mechanism.

**TABLE 1:** ACMG/AMP classification criterion: Pathogenic Strong (PS3): Well-established *in vitro* or *in vivo* functional studies supportive of a damaging effect on the gene or gene product; Pathogenic moderate 2 (PM2) = Absent from controls in Exome Sequencing Project, 1000 Genomes or ExAC; Pathogenic supporting 2 (PP2) = Missense variant in a gene that has a low rate of benign missense variation and where missense variants are a common mechanism of disease; Pathogenic supporting 3 (PP3) = Multiple lines of computational evidence support a deleterious effect on the gene or gene product (conservation, evolutionary, splicing impact, etc); Benign stand-alone 1 (BA1) = Allele frequency > 5% in Exome Sequencing, 1000 Genomes or ExAC; Benign supporting 4 (BP4) = Multiple lines of computational evidence suggest no impact on gene or gene product (conservation, evolutionary, splicing impact, etc).

Details of the variant			Minor allele frequency				Pathogenicity prediction — *in silico*				ACMG attributes	Variant classification
		
Genomic co-ordinates	AAChange. refGene	Conseq -uence	ExAC_ ALL	1000g 2015 aug_all	gnomAD_ exome_ ALL	gnomAD_ genome_ ALL	SIFT_ pred	Polyphen2_ HDIV_pred	Mutation Taster_pred	CADD_ phred		
chr2:188994587 G > A	NM_000090.3 (COL3A1_i001): p.(Gly447Asp)	Missense	.	.	.	.	D	D	D	26.1	PS3 PM2 PP2 PP3	Likely Pathogenic (II)
chr2:188999354 G > A	NM_000090.3 (COL3A1_i001): p.(Ala698Thr)	Missense	0.3204	0.21845	0.2497	0.2118	T	B	P	22.9	BA1 BP4	Benign (I)
chr2:189010695 T > G	NM_000090.3 (COL3A1_i001): p.(His1353Gln)	Missense	0.9985	0.996605	0.9988	0.9958	T	B	P	6.799	BA1 BP4	Benign (I)

AA, Amino Acid; ExAC, Exome Aggregation Consortium; 1000g, 1000 Genomes project; gnomAD, Genome Aggregation Database; SIFT, Sorting Intolerant from Tolerant; pred, prediction software; PolyPhen2_HDIV, Polymorphism Phenotyping v2 Hum Div Model; CADD_phred, Combined Annotation Dependent Depletion_phred software; D, Deleterious; PD, Probable Damaging; VUS, Variant of uncertain significance; MAF, Minor allele frequency (z score); ACMG, American College of Medical Genetics and Genomics; AMP, Association of Molecular Pathology.

According to the ACMG/AMP standards and guidelines for interpretation of sequence variants (6), the following *COL3A1* variant criteria may be used to classify exon19:c. 1340G > A variant as pathogenic variant:

PS3 → functional studies supporting damaged or altered gene product, absence of COL3A1 protein expression on immunofluorescence studies on skin fibroblasts and altered morphology of fibroblasts on electron microscopy studies.

PM2 → variant not reported before in Exome sequencing project 1000 genomes and ExAC.

PP2 → missense z-scores of COL3A1 gene > 3.09, which was regarded as intolerant to missense variants.

PP3 → SIFT (Deleterious), PolyPhen (Damaging) and a CADD-Phred score of 26.1 (pathogenic) support a deleterious effect on the gene or gene product.

Using the ACMG/AMP rules for combining the above criteria (6), *COL3A1 exon19:c.1340G* > *A* variant classification was found to be likely pathogenic (II) (Table 1).

### Treatment and management

Diagnosis and management of a rare, life threatening vascular disease condition in absence of any curative therapy is a difficult conundrum for both the patient and the treating physician. Besides symptomatic treatment and regular follow up, psychological therapy and family support is required to accept and live with such a condition. Clinical evaluation of vEDS patients includes regular blood pressure monitoring, non-invasive arterial screening to detect dissections and dilatations such as ultrasonography, computed tomography or magnetic resonance imaging (1, 2). Any invasive procedures like colonoscopy, angiograms or surgeries should be carefully assessed for risk and if necessary should be performed by surgeons experienced and familiar with the enhanced caution required due to tissue fragility and its associated complications (24).

After the confirmation of *COL3A1* variant by exome sequencing and biochemical analysis, diagnosis was confirmed as vEDS. Patient was explained about the disease and its prognosis and referred for genetic counseling. It has been reported that Celiprolol improves the biomechanical integrity of the aorta (25) and may be an option for the prevention of complications in vEDS (26). Due to lack of Celiprolol and any other approved therapies in India and many other countries, there are not many options available for treating vEDS patient besides symptomatic and supportive therapy.

As research advances further in this field, gene therapy may be a promising option for the treatment of vEDS in the future. However, addition of the defective gene is not applicable for dominant diseases such as vEDS where the defective procollagen gene, COL3A, is a homotrimer of three identical α1 chains. As most of the patients present with a heterozygous mutation, there is a structural defect in half of the synthesized collagen α1 fibrils which results in 1/8th normal and 7/8th abnormal COL3A trimers. Interesting approaches such as siRNA mediated inhibition of the mutated allele and strategies to enhance transcriptional activation of the normal allele are being tested *in vivo* for phenotypic correction of vEDS (27, 28).

A recent study has identified PLC/IP3/PKC/ERK pathway (phospholipase C/inositol 1,4,5-triphosphate/protein kinase C/extracellular signal-regulated kinase) as major drivers of vascular pathology in vEDS based on preclinical models (29). AR101 (enzastaurin) is an orally active, small molecule, serine/threonine kinase inhibitor of the PLC/IP3/PKC/ERK pathways which has been previously studied in over 40 human trials including a range of cancers.^1^ PREVEnt trial, which will assess the efficacy of enzastaurin in preventing cardiac or arterial events in patients with vEDS confirmed with *COL3A1* gene mutations is scheduled to begin next year and if successful may provide us with a new treatment for the life-threatening complications of vEDS.

## Conclusion

We evaluated a rare *COL3A1* variant in vEDS using the ACMG/AMP standards and guidelines. The diagnosis was confirmed by genetic testing and pathogenicity was established by clinical fibroblast testing and functional assays. Based on our observations, *COL3A1 exon19:c.1340G* > *A* variant may now be classified as a likely pathogenic variant for vEDS exome analysis. Further detailed study may be required to understand the exact molecular mechanism of vEDS pathogenesis in this variant.

## Data availability statement

The original contributions presented in this study are included in the article/Supplementary material, further inquiries can be directed to the corresponding author.

## Ethics statement

Ethical review and approval was not required for the study on human participants in accordance with the local legislation and institutional requirements. The patients/participants provided their written informed consent to participate in this case study. Written informed consent was obtained from the individual(s) for the publication of this case study and any potentially identifiable images or data included in this article.

## Author contributions

JM, LL, and UK conceived and designed the study. LL and UK performed the clinical evaluation. JM, AS, and SS performed and analyzed the experiments related to genetic testing and biochemistry. SG analyzed the radiology findings. JM, LL, and AS wrote the article. All authors proofread the manuscript.

## Footnotes

1 Prevention of Rupture with Enzastaurin in Vascular Ehlers-Danlos Syndrome (https://clinicaltrials.gov/ct2/show/NCT05463679).

## Supplementary material

Supplementary Table 1 Laboratory investigations.

Datasheet 1 Supplementary Data 1 (Whole exome sequencing data analysis pipeline).

Datasheet 2 Supplementary Data 2 (Materials and Methods).
