# Supplementary material for: Case report: Characterization of a rare pathogenic variant associated with loss of COL3A1 expression in vascular Ehlers Danlos syndrome
Source: Front Cardiovasc Med. 2022 Oct 11;9:939013. doi: 10.3389/fcvm.2022.939013 (PMC9595653; doi:10.3389/fcvm.2022.939013)
Supplement: Supplementary Table 1 — Laboratory investigations. [file Table_1.pdf]

Supplementary Table 1. Laboratory Investigations*

Variables	Reference Range ^	Observed
Hemoglobulin (g/dl)	13-17	14.1
Platelet count (per micro L)	150,000-400,000	231000
White-cell count (per micro L)	4000-13000	11400
Differential count (%)		
Neutrophils	40-80	65
Lymphocytes	20-40	30
Monocytes	2-10	4
Eosinophils	1-6	1
Basophils	0-3	0
Erythrocyte sedimentation rate(mm/hr)	0-13	12
Glucose (mg/dl)	70-110	85
Blood Urea (mg/dl)	19-43	19.3
Creatinine (mg/dl)	0.6-1.2	0.7
Total Bilirubin (mg/dl)	0.3-1.2	0.4
Direct Bilirubin (mg/dl)	0-0.5	0.2
Alkaline phosphatase (IU/L)	40-150	85
Aspartate aminotransaminase (IU/L)	0-42	25
Alanine aminotransaminase (IU/L)	0-60	18
Albumin (g/dl)	3.5-5.0	4
Globulin (g/dl)	2.3-3.7	2.8
Protein (g/dl)	6.3-8.2	6.8
C-reactive protein (mg/L)	0-10	**13.24**

*To convert the values for urea nitrogen to millimoles per liter , multiple by 0.357.

To convert the values for creatinine to millimoles per liter , multiple by 88.4.

To convert the values for glucose to millimoles per liter , multiple by 0.05551.

^Reference values are affected by multiple variables like population age and laboratory methods used. The ranges used are for non pregnant adults only.
