# Supplementary material for: Case report: Characterization of a rare pathogenic variant associated with loss of COL3A1 expression in vascular Ehlers Danlos syndrome
Source: Front Cardiovasc Med. 2022 Oct 11;9:939013. doi: 10.3389/fcvm.2022.939013 (PMC9595653; doi:10.3389/fcvm.2022.939013)
Supplement: Datasheet 1 — Supplementary Data 1 (Whole exome sequencing data analysis pipeline). [file Data_Sheet_1.PDF]

Whole Exome Sequencing (WES) data analysis pipeline

The fastq files from the sequencer was trimmed and the adapters were removed using Trimmomatic-0.36 maintaining an overall Phred score of 20 and a Phred score of 20 in a sliding window of 5. The quality of the trimmed data was verified using fastQC. Stampy with Burrows-Wheeler-Alignment was used for mapping of the reads to the reference genome hg38. The alignment quality was verified using Qualimap. The aligned reads were sorted using SAMtools, reads aligning to multiple loci were removed using Picard, and variant calling was performed with Platypus. BCFTools was used to screen the vcf files for depth of sequencing (depth of 10 with minimum 5 variants). The reads were visualised with Integrated Genome Viewer. Annotation of the variants was performed using ANNOVAR.
The variants were first filtered by excluding the intronic variants, followed by a selection of non-synonymous mutations. Next, frequency filters were applied with a cut-off of minor-allele frequency < 5% using ExAc and 1000 genome project databases. In-silico pathogenicity assessment of the mis-sense and indels was performed by SIFT (< 0.05), Polyphen2 (selecting ‘pathogenic’ and ‘deleterious’ variants), and MutationTaster (selecting ‘disease causing’ and ‘disease causing automatic’ variants) predictions. Finally, the variants in the genes associated with connective tissues were assessed for the presence of the filtered variants.

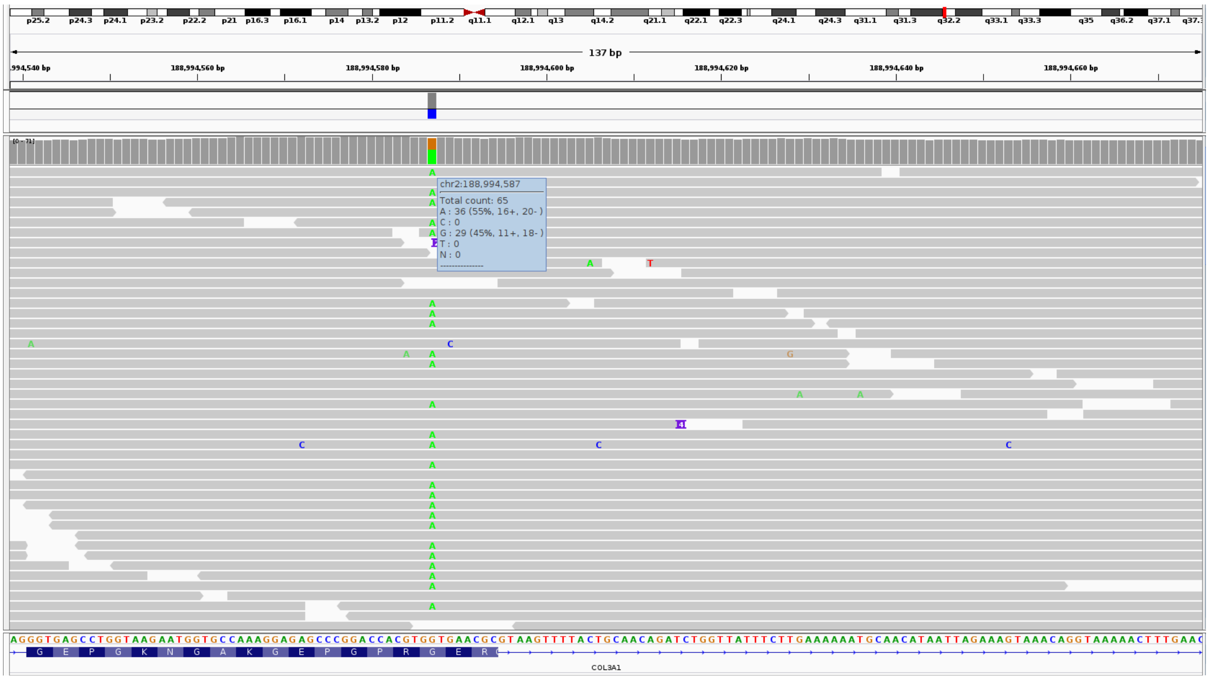

Supplementary Figure 1: Visualisation of Exon 19 of COL3A1 gene in Integrated Genome Viewer showing heterozygous c.G1340A variant, leading to p.G447D missense substitution in the COL3A1 protein.
