# Supplementary material for: Case report: Characterization of a rare pathogenic variant associated with loss of COL3A1 expression in vascular Ehlers Danlos syndrome
Source: Front Cardiovasc Med. 2022 Oct 11;9:939013. doi: 10.3389/fcvm.2022.939013 (PMC9595653; doi:10.3389/fcvm.2022.939013)
Supplement: Datasheet 2 — Supplementary Data 2 (Materials and Methods). [file Data_Sheet_2.pdf]

MATERIALS & METHODS:

Dermal Fibroblast Culture
Skin punch biopsy from the forearm of the patient and control (age and sex matched) were taken under aseptic conditions. Dermal Fibroblast explant culture was established for both the patient and control samples in our lab using standard protocols[7]. Fibroblasts were observed to grow out from the cut edges of the skin biopsy pieces 7-10 days after the outgrowth of keratinocytes.

Characterisation of fibroblasts by Immunofluorescence
Fibroblasts were fixed with ice cold 100% Methanol for 20 min at -20^oC followed by blocking with 1% Bovine Serum Albumin (BSA) and 0.5% Triton-X for 1 hour at room temperature. Cells were incubated with Primary Rabbit anti human-COL3A1 antibody,1:200 (Abcam Cat# ab6310) and Primary Mouse anti human-beta tubulin antibody, 1:400 (Sigma Cat# T8328) in blocking buffer for 1 hour at room temperature. This was followed by incubation with secondary Donkey anti-rabbit conjugated to NorthernLights-557, 1:500 (R&D systems Cat#NL004) and Goat anti-mouse Alexa Flour 488, 1:500 (Invitrogen Cat# A-11001) for 1 hour at room temperature in the dark followed by incubation with DAPI (4′,6-diamidino-2-phenylindole) for 1-2 minutes in the dark at room temperature. Immunofluorescent signals were obtained on a Nikon A1 confocal Laser Microscope. All experiments were repeated three times.

Transmission electron microscopy (TEM) of Fibroblasts
The fibroblast cell pellet was washed with 0.1 M phosphate buffer (PB) and fixed in a mixture of 2% glutaraldehyde and 2% paraformaldehyde for 2 hours at 4^oC. Pellet was washed in PB and postfixed in 1% osmium tetroxide at 4^oC for 1 hour. This was followed dehydration in acetone, infiltration and embedding in araldite CY212 (TAAB, UK) and sectioning using ultramicrotome. These 1 µm sections were mounted on glass slides, stained with aqueous toluidine blue and observed under a light microscope for gross observation. For electron microscopy, thin sections (70-80 nm) were cut and mounted onto 300 mesh-copper grids. Sections were stained with alcoholic uranyl acetate and alkaline lead citrate, washed gently with distilled water and observed under a Morgagni 268D transmission electron microscope (Fei Company, The Netherlands) at an operating voltage of 80 kV. Images were digitally acquired by using a CCD camera (Megaview III, Fei Company) attached to the microscope.

DNA Extraction
Genomic DNA was isolated from peripheral blood and from cultured fibroblasts using QIAamp DNA mini kit (Cat no. 51304 Qiagen, Netherlands). The quality of isolated DNA was checked using agarose gel electrophoresis and nano-spectrophotometer. The DNA was stored at -20 °C until further analyses.

Whole Exome Sequencing (WES)
5 µg of genomic DNA isolated from the peripheral blood was used for preparation of DNA libraries according to the manufacturer’s protocol (Agilent Technologies Inc., Santa Clara, CA, USA). The libraries were prepared using Agilent Sure Select Human All Exon v7 kit for paired end Illumina sequencing using Illumina Hiseq2000 as per the standard protocols. 2 x 150 bp sequencing was performed to achieve an average sequencing depth of 100X. Refer to Supplementary data 1 for details on data analysis.

Sanger Sequencing
Genomic DNA extracted from cultured skin fibroblasts was subjected to polymerase chain reaction, using Phusion high fidelity DNA polymerase (Cat# F530S Thermo Scientific, USA) for 35 cycles, with primers flanking the variant; forward primer

5'TGCTAATGGTGCTCCTGGAC3' and reverse primer 5'GGAATACCAGCCTCACCCTG3'. The amplicon obtained was run on an agarose gel, followed by the elution using QIAquick gel extraction kit (Cat# 28704 Qiagen, Netherlands). The eluted amplicon was Sanger sequenced and the sequences obtained were aligned to the Ensembl human gene sequence using EMBOSS Water nucleotide alignment for confirmation of the variant.
